# Identifying Self-Management Support Needs for Pregnant Women With Opioid Misuse in Online Health Communities: Mixed Methods Analysis of Web Posts

**DOI:** 10.2196/18296

**Published:** 2021-02-04

**Authors:** Ou Stella Liang, Yunan Chen, David S Bennett, Christopher C Yang

**Affiliations:** 1 College of Computing and Informatics Drexel University Philadelphia, PA United States; 2 Donald Bren School of Information and Computer Sciences University of California, Irvine Irvine, CA United States; 3 Department of Psychiatry College of Medicine Drexel University Philadelphia, PA United States

**Keywords:** self-management, online health community, opioid use disorder, pregnancy

## Abstract

**Background:**

The current opioid crisis in the United States impacts broad population groups, including pregnant women. Opioid use during pregnancy can affect the health and wellness of both mothers and their infants. Understanding women’s efforts to self-manage opioid use or misuse in pregnancy is needed to identify intervention points for improving maternal outcomes.

**Objective:**

This study aims to identify the characteristics of women in an online health community (OHC) with opioid use or misuse during pregnancy and the self-management support needs of these mothers.

**Methods:**

A total of 200 web posts by pregnant women with opioid use participating in an OHC were double coded. Concepts and their thematic connections were identified through an inductive process until theoretical saturation was reached. Statistical tests were performed to identify patterns.

**Results:**

The majority of pregnant women (150/200, 75.0%) in the OHC exhibited signs of misuse, and 62.5% (125/200) of the participants were either contemplating or pursuing dosage reduction. Self-managed withdrawal was more common (*P*<.001) than professional treatment among the population. A total of 5 themes of self-management support needs were identified as women sought information about the potential adverse effects of gestational opioid use, protocols for self-managed withdrawal, pain management safety during pregnancy, hospital policies and legal procedures related to child protection, and strategies for navigating offline support systems. In addition, 58.5% (117/200) of the pregnant women expressed negative emotions, of whom only 10.2% (12/117) sought to address their emotional needs with the help of the OHC.

**Conclusions:**

OHCs provide vital self-management support for pregnant women with opioid use or misuse. Women pursuing self-managed dosage reduction are prone to misinformation and repeated relapses, which can result in extreme measures to avoid testing positive for drug use at labor. The study findings provide evidence for public policy considerations, including universal screening of substance use for pregnant women, emphasis on treatment rather than legal punishment, and further expansion of the Drug Addiction Treatment Act waiver training program. The improvement of web-based platforms that can organize geo-relevant information, dispense clinically validated withdrawal schedules, and offer structured peer support is envisioned for harm reduction among pregnant women who opt for self-management of opioid misuse.

## Introduction

### Background

I literally feel like this is the end. Like there is no way out. ...I don't want the heroin to harm the baby and I don't want withdrawals to harm the baby. And I don't have the money...

P1311

This is a quote from a pregnant woman in an online health community (OHC) who shared her dire circumstances. She is one of an estimated 21,000 pregnant women who reported misuse of opioids in the past month in the United States [1]. In a recent Centers for Disease Control and Prevention study, 6.6% of women reported using prescription opioids during pregnancy, among whom 21.2% reported misuse (ie, illicit use or nonmedical use of prescription opioids) [2]. Pregnant women who misuse opioids face obstetric complications, and their infants are at risk of developing neonatal abstinence syndrome (NAS), which can impede neonatal growth and increase the risk of other physical and developmental problems [3].

### Study Aims

Opioid use during pregnancy is heavily stigmatized and may be legally prosecuted in many states [4,5]. Therefore, we hypothesize that women may choose to self-manage their drug use without the assistance of a health care provider, and as a result, little is known from clinical data about their health challenges and behaviors. To better understand their experiences, we examine data from an OHC, where women seek help, in part, because of the community’s anonymity. This study aims to identify (1) the characteristics of women in a public OHC who self-manage their opioid use during pregnancy and (2) their self-management support needs by qualitatively analyzing personal narratives posted in the community. In doing so, we hope to identify intervention points at the policy, organizational, and technology levels that can support the unique needs of this population and achieve harm reduction.

Next, we will briefly review the related literature on the treatment of opioid misuse during pregnancy, barriers to care for pregnant women with opioid use, self-management support, and OHCs.

### Treatment for Opioid Misuse During Pregnancy

Research on the best clinical practice for managing opioid use disorder (OUD) in pregnancy has significantly advanced in the past two decades [6-8]. The American College of Obstetricians and Gynecologists recommends medication-assisted treatment (MAT) over medically supervised withdrawal (because of high relapse rates associated with withdrawal), modified prenatal care elements for OUD-related health needs, and postpartum psychosocial support services [9]. Among MAT medications, buprenorphine may result in better neonatal outcomes than methadone, including higher birth weights and lower treatment times for NAS [10]. Definitions related to OUD and treatment can be found in Multimedia Appendix 1 [8-14].

### Barriers to Care for Pregnant Women With Opioid Use

Survey studies and interview-based qualitative analyses have provided a contextual understanding of the barriers to care, including prenatal care and substance use treatment, encountered by pregnant women with opioid misuse. They are often of low socioeconomic status and experience significant obstacles such as lack of insurance coverage and transportation to access prenatal care, with their opioid use making it more difficult to resolve these barriers [15,16]. With regard to seeking substance use treatment in pregnancy, fear of losing child custody and concerns about being stigmatized are cited by pregnant women as barriers [17].

### Self-Management Support

Health agencies in the United States and across the world have recently recognized the importance of facilitating patients’ self-efficacy in managing chronic conditions. *Self-management support* focuses on empowering individuals to take active steps in managing their own chronic conditions by providing the necessary skills and confidence via interventions such as physical activities, education, and peer support [18-20]. Studies have demonstrated the effectiveness of self-management support programs, to an extent, for managing long-term conditions such as diabetes, heart failure, dementia, and Parkinson disease [21-27].

There is evidence that self-management support interventions alongside standard care can be effective for severe mental health problems [28,29], although specific interventions for substance use disorders (SUD) have not been designed or studied [30]. A search on *self-management support for substance use disorder* on PubMed yielded few relevant results at the time of writing (November 2020). Brown and Altice [31] studied themes related to self-management of MAT medications and found that online participants discussed personal experience and strategies of using unprescribed medication, distrust with health care providers, and desire to recover. Schaub et al [32] demonstrated the effectiveness of a web-based self-help intervention for participants with problematic cannabis use if it can be supplemented by brief chat counseling.

### OHCs

Multiple studies point to the utility of web-based interventions in the negotiation of self-management work [27,29,33-35]. A meta-analysis found 4 mechanisms of self-management support in online groups: collective knowledge and identity building through lived experience, social support through readily accessible gifting relationships, sociability beyond illness, and online disinhibition [35]. Here, we view OHCs in their broadest sense: they are self-organizing web-based interest groups voluntarily joined by patients, caretakers, and sometimes health professionals with a shared interest in similar health conditions. OHCs can exist in dedicated forums, such as PatientsLikeMe [36] and BabyCenter [37]; in mobile health apps, such as fertility and dietary tracking apps; or on general-purpose social media platforms, such as Facebook and Reddit. They can be created and governed by health care organizations, technology companies, or patient advocacy groups.

The majority (61%) of US adults use the internet to find health information [38]. Health consumers turn to OHCs, where participants exercise collective sense making to process competing online viewpoints [39,40], to gain experiential expertise from peers that their physicians may not provide [41-44], and to formulate actionable insights for health management [44-46]. Pregnant women seek peer support from OHCs because of constrained access to health care, dissatisfaction with care received, limited offline support, and the unavailability of information from other venues [47]. In addition to information dissemination, several studies point to OHC’s utility in enhancing human connection [48-51] and in helping participants overcome stigma [49,52]. Social ties formed in online groups provide space for self-management work that can improve the experience of participants with long-term illnesses [35]. As such, OHCs provide a suitable setting by which we can obtain an initial understanding of people’s experiences in sensitive and stigmatized situations, capturing not only their circumstances but also their emotional states [42,45,53]. The disadvantages of OHCs, however, include their lack of quality assurance on the consumer health information provided [54], a lack of recognized credibility from health care providers [55], and possible reinforcement of negative behaviors among people in the same network [56].

## Methods

### Data Source

We analyzed participant-generated content from an OHC (name omitted for the protection of user privacy) that has a long-standing history and active user participation. Compared with other social media platforms, the OHC (1) is anonymous, allowing for discussion of stigmatized and sensitive health topics; (2) does not have length limits, thereby providing space for relatively detailed accounts of personal experiences; (3) has a wide range of coverage in health condition topics, including pregnancy, substance use, and pain management, so that participants are not constrained to discuss only one aspect of their health given the complex nature of gestational opioid use; and (4) has a long history that allows us to study the activities of OHC participants at the beginning of the millennium when reports of overdoses from prescribed opioids began to rise sharply [57].

### Ethics and Privacy Protection

OHC content is considered public and exempt by institutional review boards as publicly available social media data per the Code of Federal Regulations (CFR) Title 45 Part 46.101 Paragraph (b) Categories of exempt human subject research (4) [58]. We took precautions in handling the OHC data because of its sensitive nature [59,60]. The data set was deidentified by removing users’ screen names and assigning a randomly generated identifier number independent of the OHC. In reporting the findings, quoted sentences were removed from potentially sensitive personal information (eg, state of residence), misspellings were corrected to help mask linguistic identity, and recounts of events were paraphrased.

### Data Collection

We queried the said OHC for posts made from 2000 to 2019 that contain a *pregnancy concept equivalent* and at least one OUD-related drug name. A set of pregnancy concept equivalents, namely, “pregnant, pregnancy, expecting, baby, infant, fetus, preggers, and preggy”, was iteratively developed by incorporating common expressions in sample posts and synonyms to the term *pregnant*. A list of drugs was built with the generic and brand names of the top 10 prescribed opioids among commercially insured pregnant women in the United States [61] and commonly abused prescription opioids and heroin listed by the National Institute on Drug Abuse [11]. Opioid antagonists as MAT medications were also included. The list of 36 drugs can be found in Multimedia Appendix 2. Due to the nature of the string-matching query, false-positive posts were discarded during the coding process. For example, nonpregnant SUD recovery participants may use the word *expecting* in the context of anticipating an outcome.

### Data Analysis

The unit of analysis is an initiating post that refers to pregnancy and opioids and does not include its comments. We performed an inductive thematic analysis on the qualifying posts following the procedures outlined by Braun and Clarke [62]. An inductive approach is driven by data without forcing emerging themes to a pre-existing coding framework [62]. The sample size was determined by the saturation principle, namely, coding was conducted until additional samples yielded no additional insights into the topic of research [63].

Two researchers (a doctoral student with a public health background and a master’s student with a nursing background) iteratively annotated the same set of 200 randomly sampled posts divided into 3 coding runs. We first annotated 100 posts and recorded all appearing concepts, which were then grouped into key themes to form a codebook. For example, upon seeing many posts that described efforts of reducing the opioid dosage, we created the themes *opioid experience*, *trimester*, *recovery stage*, and *recovery method*, as it was clear that women usually mentioned their opioid experience and stage of gestation as a context for discussing the recovery methods and their recovery progress. Second, we annotated 50 additional posts and refined the codebook by placing similar concepts together. For example, inquiries on neonatal withdrawal were combined with general questions about the drug safety of opioids as *adverse effects of gestational opioid use*. Third, we annotated another 50 posts to confirm that no new concepts emerged from the annotation to reach saturation. The development of the codebook was supervised by an experienced researcher in human-computer interaction. The definitions and exemplary quotes of the key themes and concepts are provided in Table 1. Among the variables, up to 3 (the maximum number found in the posts) emotions and self-management support needs were annotated, as participants can express more than one concept (emotion or concern) in the same post. To measure interrater reliability, the annotations have a Cohen kappa of 0.863, which suggests a high level of agreement. Interpretative differences were discussed among the 3 researchers and resolved.

**Table 1:** Codebook.

Study aim, theme, and concept definition			Example	
**Study aim 1**				
	**Opioid experience**			
		Opioid naïve: Temporary use of opioid prescriptions for acute pain lasting fewer than 3 months		“I had stomach pains and went to the emergency room. The doctor gave me morphine.” [P3660]
		Opioid misuse: Meeting one or more DSM-V^a^ diagnostic criteria for OUD^b^ [64] Receiving treatment for OUD		“I have been self-medicating myself a total of 18.75 mg daily for about a year and a half. When I stopped, I experienced withdrawal symptoms! Pretty intense ones too.” [P90] “I’m currently on methadone for an opiate addiction.” [P1600]
		Unable to determine		“I am 3 weeks pregnant and on Norco, Flexeril, Xanax. I have been taking them for 2 years.” [P1830]
	**Recovery stage**			
		Precontemplation: No mention of interest in reducing the opioid dosage		“I’ve been taking Vicodin and Percocet and I’m 20 weeks pregnant.” [P5357]
		Contemplation: Expressing interest in reducing the dosage		“I am ready and would like to quit the suboxone cold turkey.” [P4191]
		Action: Describing experience during withdrawal or relapse		“I am on day 2 of detoxification. How long till I feel better?” [P4074]
		No misuse		“I am 38 weeks pregnant and my Ob prescribed me Percocet for kidney stones. I passed the stone today, so I won’t be needing the pain killers anymore.” [P6315]
	**Recovery method**			
		Tapered withdrawal (self-managed): Describing preference for gradually reducing the dosage of opioids		“I have been detoxing for 2 months and have gone down to 29 mgs.” [P1649]
		Sudden discontinuation (self-managed): Using expressions that indicate full discontinuation of opioids		“I have 2 weeks left till my delivery date I stopped taking the Norco today.” [P1339]
		Undecided self-recovery: Not specifying a particular method but expressing interest in dosage reduction on one’s own		“I know I can’t do this cold turkey and am unsure of my will power to taper.” [P6576]
		Professional treatment: Receiving substance use treatment from professionals		“I take the methadone daily at a clinic near where I live.” [P1600]
		MAT^c^ (source unknown): Using MAT medications from unspecified sources		“Is it safe to use Suboxone while being pregnant?” [P6066]
		Not applicable: Women who did not have opioid misuse or not pursuing recovery		“I’m 27 weeks pregnant and have peed blood, and my sides where my kidneys are have been hurting really bad. I’m trying not to take the Tylenol with codeine, but I might have to if it keeps getting worse.” [P17]
	**Trimester**			
		First trimester: 0-13 weeks		“I have just found out I am 6 weeks pregnant.” [P6434]
		Second trimester: 14-27 weeks		“I am currently 22 weeks pregnant with my second child.” [P3644]
		Third trimester: 28+ weeks		“I am 39 weeks pregnant.” [P5896]
		Unspecified		“I think I’m on the right path now, but I’m scared it will still be in my system when I have the baby.” [P460]
**Study aim 2**				
	**Self-management support needs—informational**			
		Potential adverse effects of gestational opioid use Neonatal withdrawal Open-ended inquiry		“I was just wondering if my baby could possibly withdrawal from these?” [P6342] “Has anyone taken this and (was) baby ok?” [P217]
		Self-managed withdrawal		“Has anyone had any good stories with getting lowered slowly off it?” [P12765]
		Pain management safe for pregnancy		“I am prescribed Percocet for pain. Is it bad for baby, is there a best alternative?” [P1283]
		Legal procedures		“I need to know if they can take my baby because of this prescription showing up?” [P5143]
		Navigating offline support systems: Looking for recommendations of treatment facilities Interacting with providers Interacting with caretakers		“If anyone knows of an OBGYN that takes [insurance name] and is familiar with my situation, or a pain management doc that deals with pregnancies and also takes my insurance...just SOMEWHERE to start.” [P141] “I’m not sure what to do anymore. I don’t want my doc to think I’m abusing them or selling then because I swear I’m not!” [P8] “I just don’t want to bring up my drug use to my mom because I know it’s going to hurt her. I don’t know what to do or what to say that doesn’t have my mom worry about my baby’s healthy.” [P3413]
		Other pregnancy concerns		“I am not sure if I have felt the baby move yet, I am 18 weeks today. Is that ok?” [P6689]
	**Appropriateness of tapering schedule**			
		Appropriate: The tapering plan is consistent with clinical guidelines		No instances were found.
		Inappropriate: The tapering plan is too rapid		“I took 3, 30 mg for the past few days and I took 2, 30 mg today. I am going to take 1, 30 mg tomorrow, 15 the next and half that the following.” [P6844]
		Unclear: Not enough information to determine appropriateness		“Can I ween myself off of Methadone slowly, very slowly?” [P3391]
	**Self-management support needs—emotional**			
		Seeking emotional support		“Some days I have a hard time staying positive. Please if anyone is available to talk with me, I would really appreciate it.” [P3899]
	**Sentiment**			
		Negative sentiments: Fear Shame Anxiety Despair		“I am terrified of losing him.” [P3545] “I am very ashamed.” [P90] “I am planning to cut down and then quit because I’m worried, even though doctors say they are ok.” [P369] “I am so ashamed, scared, and lonely! I feel hopeless!” [P4608]
		Mixed sentiment: Cautious optimistic		“I feel much more optimistic about beating my addiction for good after coming to this forum. It’s hard when you have no one to talk to or share experiences with.” [P2137]
		Positive sentiment: Positive emotions, such as hope and love		No instances were found.

^a^DSM-V: Diagnostic and Statistical Manual of Mental Disorders, Fifth Edition.

^b^OUD: opioid use disorder.

^c^MAT: medication-assisted treatment.

Furthermore, women’s common beliefs were compared with the scientific literature and clinical guidelines to discern any divergence. An experienced clinical psychologist annotated whether misconception existed compared with clinical guidelines in the self-guided withdrawal plans described by participants [65].

### Data Reporting

Themes from inductive coding were grouped in the results to address the 2 study aims. First, the characteristics of OHC participants (study aim 1) are represented by themes coded under *Opioid experience*, *Recovery stage*, *Recovery method*, and *Trimester*. Second, the self-management support needs (study aim 2) are represented by concepts coded under self-management support *needs—informational* and self-management support *needs—emotional* and complemented by the themes *Appropriateness of tapering schedule* and *Sentiment*.

## Results

### Metadata

The search query yielded 3559 posts between 2000 and 2019. Posts appeared in 201 subgroups related to substance misuse (2096/3559, 58.89%), pregnancy (680/3559, 19.11%), and others (783/3559, 22.00%), such as neurology and back pain. The mean number of drug names mentioned per post was 1.3 (SD 0.7). The mean character count per post was 1411.2 (SD 1313.7).

### Study Aim 1: Characteristics of Pregnant Women With Opioid Use in OHC

The majority (150/200, 75.0%) of women who took opioids during pregnancy in the OHC (*the study population*) met one or more criteria from the Diagnostic and Statistical Manual of Mental Disorders, Fifth Edition [64] for a potential OUD diagnosis, which we refer to as *opioid misuse* as we were unable to make a formal clinical diagnosis. Close to half (94/200, 47.0%) of the study population were in the process of pursuing dosage reduction (ie, action stage), with another 31 participants (31/200, 15.5%) considering but not yet initiating a reduction in dosage (ie, contemplation stage; Table 2). In terms of recovery method, self-managed withdrawal was more common than professional treatment (*P*<.001). This indicates that women in the OHC primarily elect to self-manage their attempts at dosage reduction during pregnancy.

**Figure 1:**
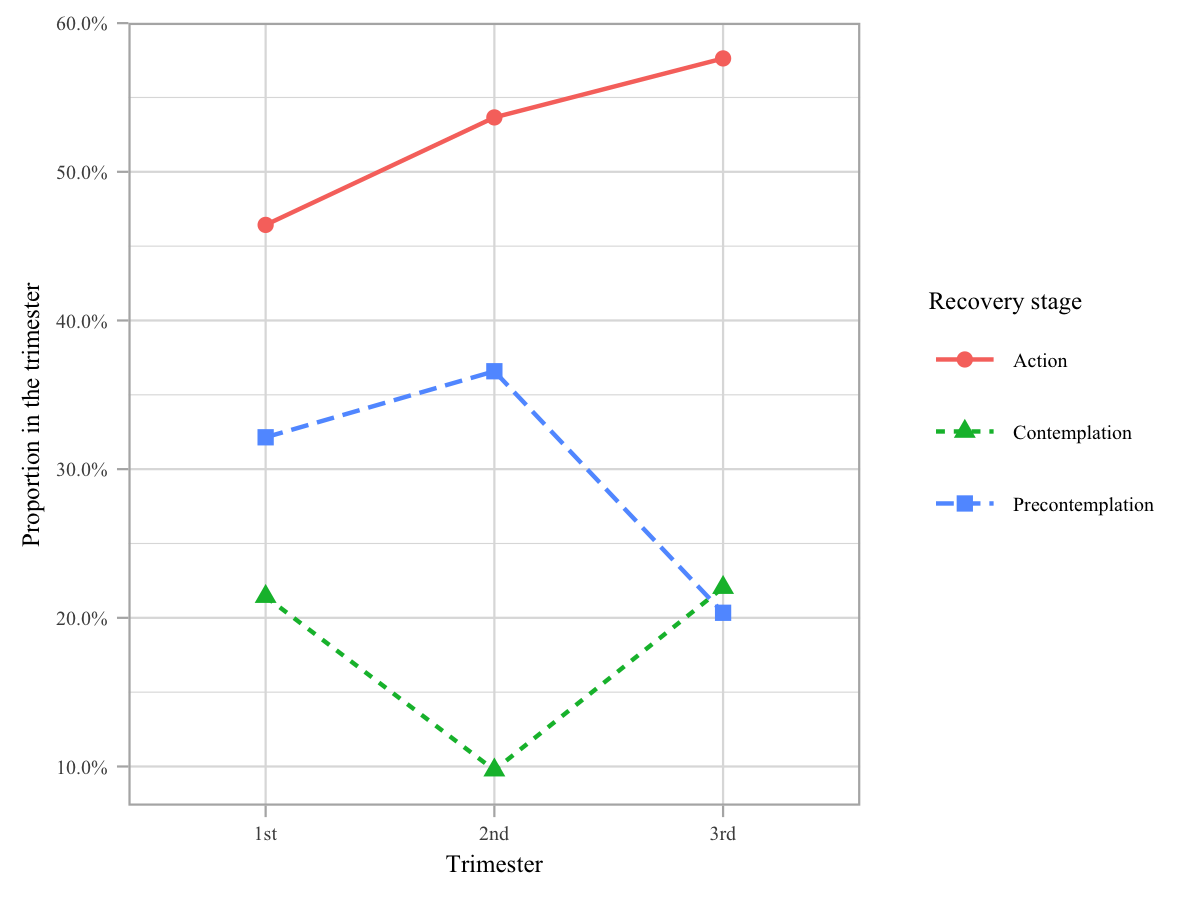
Proportions of the recovery stages by trimester.

Gestationally, the women were primarily in their first or third trimesters, and notably, the percentage of women not pursuing recovery (ie, precontemplation stage) decreased (*P*=.11) and the percentage of those in action increased (*P*=.16) from the first to the third trimester, although the difference was not statistically significant (Figure 1).

**Table 2:** Characteristics of women inquiring about opioid use in the online health communities (N=200).

Characteristic			Participants, n (%)
**Opioid experience**			
	Opioid naïve		22 (11.0)
	Opioid misuse		150 (75.0)
	Unable to determine		28 (14.0)
**Recovery stage**			
	Precontemplation		51 (25.5)
	Contemplation		31 (15.5)
	Action		94 (47.0)
	No misuse		24 (12.0)
**Recovery method**			
	**Self-managed withdrawal**		
		Tapered withdrawal	40 (20.0)
		Sudden discontinuation	35 (17.5)
		Undecided	15 (7.5)
	Professional treatment		29 (14.5)
	Medication-assisted treatment (unknown sources)		6 (3.0)
	Not applicable		75 (37.5)
**Trimester**			
	First		61 (30.5)
	Second		49 (24.5)
	Third		66 (33.0)
	Unspecified		24 (12.0)

### Study Aim 2: Self-Management Support Needs

**Figure 2:**
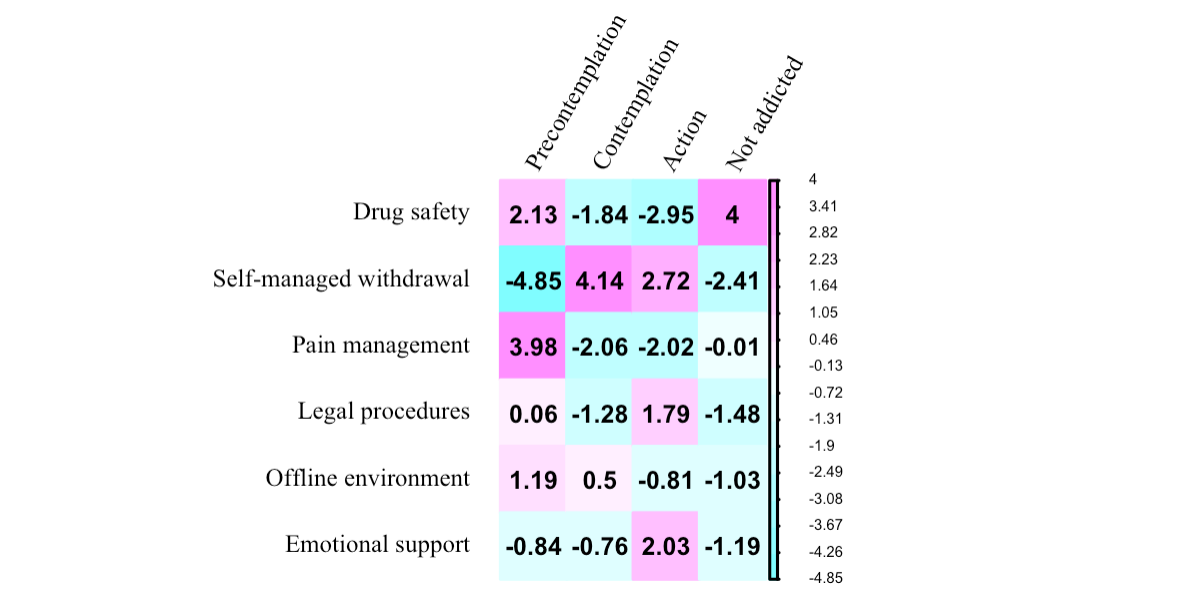
Standardized residuals of associations between self-management support needs and recovery stages.

Following thematic analysis of the web posts, 6 primary themes of self-management support needs were identified, including information needs for understanding (1) the potential adverse effects of gestational opioid use, (2) self-led withdrawal, (3) the safety of continued opioid use for pain management during pregnancy, (4) legal procedures related to child protection, (5) navigating offline health care systems, and (6) needs for emotional support (Table 3). Other pregnancy concerns and posts with unspecified support needs were excluded from further analysis because they do not pertain to opioid use. The women had different self-management support needs according to their recovery stages (*χ*^2^_15_=69.5; *P*<.001). The absolute value of a standardized residual (r_std_) greater than 2 indicates a strong (dis)association (Figure 2), on which we will elaborate next.

**Table 3:** Self-management support needs expressed in the online health community postings (N=200).

Themes and concepts		Participants, n (%)	
**Self-management support needs–informational**			
	Potential adverse effects of gestational opioid use		99 (49.5)
	Self-managed withdrawal		70 (35.0)
	Pain management safety during pregnancy		20 (10.0)
	Legal procedures		18 (9.0)
	Navigating offline support systems		9 (4.5)
**Self-management support needs–emotional**			
	Seeking emotional support		12 (6.0)
**Excluded**			
	Other pregnancy concerns		5 (2.5)
	Unspecified		4 (2.0)

#### Potential Adverse Effects of Gestational Opioid Use

The most common concern (99/200, 49.5%) of the study population was the potential adverse effects of opioids on the fetuses. In particular, the questions on drug safety have a high level of homogeneity and can be simply divided into 2 categories: general inquiry as in “was your baby okay?” [P3614] and specific inquiry on the potential of neonatal withdrawal as in “I am scared to death my baby will have withdrawals” [P16]. These concerns were primarily seen among those who did not consider treatment for opioid misuse (r_std_=2.13) or did not have a misuse (r_std_=4.00), whereas those in active pursuit of recovery were least concerned with the drug safety in their posts (r_std_=−2.95; Figure 2).

#### Self-Managed Withdrawal

Questions about how to reduce opioid dosage during pregnancy were the second most common (70/200, 35.0%), primarily among those contemplating (r_std_=4.14) or undergoing (r_std_=2.72) treatment of opioid misuse (Figure 2). These concerns included (1) comparing strategies to reduce opioid dosage, (2) discussing withdrawal schedules, (3) enduring withdrawal-related hardships, and (4) dealing with the aftermath of relapses.

First, women contemplating dosage reduction used the OHC as a sounding board to plan their course of action in the absence of professional advice. They debated the risks and benefits of withdrawal during pregnancy and how to do so safely. They were concerned about the impact on the fetus if withdrawal symptoms occurred during their pregnancy:

I'm scared about the withdrawals of MS Contin, for which I'm fully prepared, but scared it will harm my child?

P6419

They also compared sudden discontinuation to tapered withdrawal:

I considered quitting cold turkey, but I have read that it is a bad idea. Would tapering be a good idea? What is a good taper method?

P2325

Although this shows some women were aware of the disadvantages of sudden discontinuation, tapered withdrawal (40/200, 20.0%) and sudden discontinuation (35/200, 17.5%) were equally popular among the study population (*P*=.61).

Second, once committed to decreasing their opioid dosage, the women may describe a detailed tapering schedule in the OHC to solicit peer feedback:

Should I just do half of what I have been for a few days, then cut it in half again, do that for a couple days, then go for nothing?! I really don't know what to do, that's why I am asking for help.

P1628

Of all the women who indicated an interest in tapered withdrawal, only 23% (9/40) provided specific information on how they intended to do so, suggesting that most did not have a structured plan on how to taper. Furthermore, *all* of these plans were deemed too rapid (Table 4) based on clinical guidelines and could increase the risk of painful withdrawal symptoms [65].

**Table 4:** Appropriateness of tapering schedule (N=40).

Appropriateness	Participants, n (%)
Unclear	31 (78)
Inappropriate (too rapid)	9 (22)
Appropriate	0 (0)

Third, after initiating withdrawal, the women turned to the OHC for practical advice and emotional support when undergoing the hardship of withdrawal, sometimes stemming from the lack of a proper tapering schedule:

Just started detox on tramadol today. I feel like hell and my legs are killing me! I'm 26 hours into it. How much more do I need to endure?

P2423

As a result of significant withdrawal symptoms, they were prone to setbacks and relapses:

Unfortunately, I relapsed and slowly started using until now (37 weeks). I have stopped a couple of times but have had issues because of withdrawal fears.

P5992

To combat the demoralizing impact of relapses, some women looked to the OHC to hold them accountable:

I am just looking for some support, accountability and encouragement as I feel so alone, scared and terrible about my relapse and lying to my love.

P3545

These experiences highlight the absence of structure and support with self-managed withdrawal as compared with being in a clinical program where physician supervision and evidence-based therapies are provided to help patients manage their dosage reduction process more effectively.

Fourth, the repeated attempts and failures to decrease opioid use during pregnancy were not only physically taxing and emotionally draining but also meant that the women with clandestine opioid use would have no other perceived choice but to resort to sudden discontinuation when the date of delivery approached in hope of a negative drug test, despite the dangers of doing so abruptly. Women in this position asked:

How long will it take for the test to be negative if I stop today?

P5891

**Figure 3:**
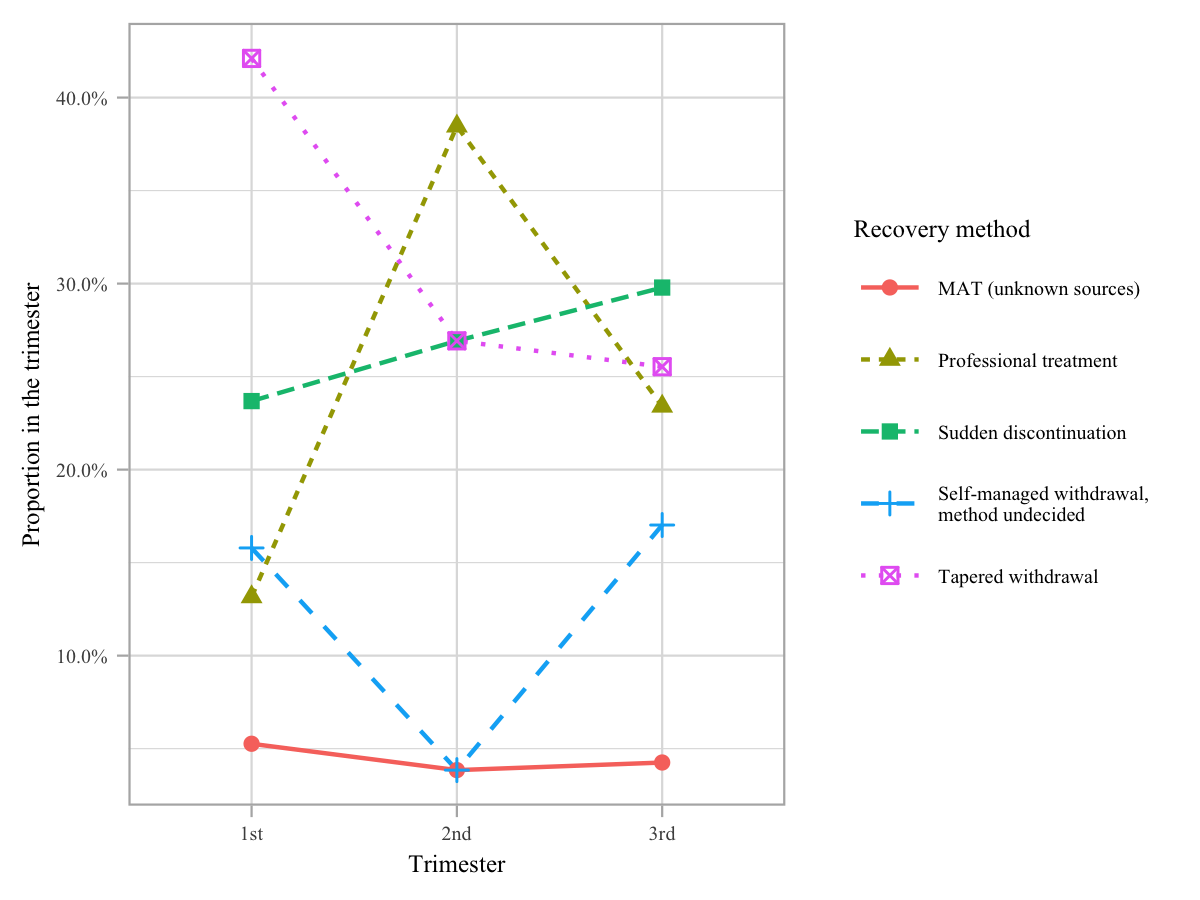
Proportions of the recovery methods by trimester.

The commonness of this risky approach is evident in the increasing percentage (*P*=.70) of women attempting sudden discontinuation of dosage and the decreasing percentage (*P*=.17) of tapered withdrawal during the third trimester (Figure 3), although the changes were not statistically significant.

#### Pain Management

Women using opioids for chronic pain management before becoming pregnant faced the dilemma of leaving their severe pain untreated or risking side effects to their fetus. They had difficulty transitioning to gestational opioid use:

I have been in pain management for 3 years due to a dislocated hip. Apparently neither my OB nor primary care doctor have ever dealt with “my situation” before.

P141

In looking for safe pain medications to take during pregnancy, they (20/200, 10.0%) viewed peers’ experience as empirical evidence and second opinions to professional advice:

I have 4 discs missing in my spine. I am now 4 months pregnant and my doctor has taken me off everything!!! Does anyone know what is safe for the baby?

P6486

As they continue to take opioids during pregnancy, concerns related to pain management are mostly associated (r_std_=3.98) with those in the precontemplation stage (Figure 2).

#### Understanding Legal Procedures

Concerns related to Child Protective Services (CPSs) procedures were present in 9.0% (18/200) of the posts. These concerns were more common among women in the recovery stage of action (r_std_=1.79) than in other stages (Figure 2). Although the regulation and laws related to child protection from drug use are obscure to laypersons, the vague idea of losing parental guardianship looms large. Pregnant women with opioid misuse in the OHC described intense fear of being identified as *using* and consequently losing parental guardianship of their newborn and the goodwill of their direct support systems:

I am scared to death of having to deal with child protection service especially since none in my family knows about any of this and the fear of my child being taken away from me.

P16

They inquired about the role of hospitals in reporting drug use instead of directly asking their providers:

Will child services still get involved? I have heard that doctors do these types of things and contact these types of people behind your back?

P3776

Notably, those who used unprescribed MAT medications to self-treat OUD (6/200, 3.0%) may still be held accountable for illegal possession and opioid use during pregnancy.

#### Navigating Offline Support Systems

A few women (9/200, 4.5%) also requested strategies for navigating their offline support systems, including recommendations for treatment facilities (4/200, 2.0%), advice for interacting with health care providers (4/200, 2.0%), and disclosing opioid use with their family members (1/200, 0.5%). These concerns were not particularly associated with one recovery stage. Accessing specialized prenatal care for women with opioid use or finding substance treatment programs that accept pregnant women presented a challenge. Locating a specialty program proved to be difficult even with an obstetrician’s referral:

My obstetrician had made me an appointment with a therapist, who tells me she doesn't even see pregnant women! I got some numbers for the methadone clinic and other places but yet again no one will help a pregnant woman.

P3855

This post was made after 2010, which is a decade after the passing of the Drug Addiction Treatment Act (DATA) of 2000, which allows trained physicians including obstetricians to treat opioid dependency with MAT medications. Women also consulted the OHC for strategies on how to best interact with their providers and caretakers to explain personal circumstances involving opioid usage and building rapport (examples are given in Table 1).

#### Seeking Emotional Support

Besides the needs for informational support, a few women (12/200, 6.0%) explicitly requested emotional support:

Please send me some words of encouragement.

P3545

In contrast, a high number of posts (117/200, 58.5%) described negative emotions, including fear, shame, anxiety, and despair (Table 5; examples are given in Table 1). In other words, only about 1 out of 10 women who experienced negative emotions sought to address their emotional needs with the help of the OHC. Importantly, expressing negative emotions did not modify the women’s likelihood of pursuing (ie, action or contemplation) dosage reduction (*χ*^2^_1_=0.1; *P*=.75).

**Table 5:** Sentiment (N=200).

Sentiment	Participants, n (%)
Negative	117 (58.5)
Not specified	82 (41.0)
Mixed	1 (0.5)
Positive	0 (0.0)

## Discussion

### Principal Findings

The majority of pregnant women in the OHC exhibited signs of opioid misuse, with approximately two-thirds of them pursuing recovery. Self-managed withdrawal of opioid use was more common than professional treatment. The following 6 identified themes highlighted women’s self-management support needs: (1) providing clarity on the impact of opioid drugs on pregnancy; (2) providing clinically validated information on how to scientifically reduce opioid dosage; (3) providing guidelines on safe pain management practice during pregnancy; (4) providing information on local CPSs procedures, including the hospital’s role in reporting; (5) providing strategies for interacting with and obtaining support from offline support systems; and (6) providing emotional support for those experiencing negative emotions.

The study population relied heavily on the OHC to provide guidance in the absence of professional care and in-person support, which differentiates them from other patient groups that usually use online support groups as a supplement to traditional health care services or in-person support groups [66,67]. Formal evaluation and proper treatment recommendations were lacking for pregnant women in the OHC who chose self-managed withdrawal. Despite their resolve to reduce opioid dosage, women were vulnerable to the pitfalls of misinformation (eg, overly aggressive tapering schedules) and could easily experience relapses that may cause distress in both the mother and fetus.

### Indirect Emotional Support

Experiencing negative emotions is commonplace for the study population, but explicitly requesting emotional support is not. Furthermore, although positive emotions are shown to be a facilitator of self-efficacy, which is a key construct in the social cognitive theory for effecting health behavior change [68,69], the opposite may not be true: experiencing negative emotions does not modify the women’s likelihood of pursuing dosage reduction. This contrast, on the one hand, shows that women with opioid use or misuse during pregnancy were preoccupied with seeking information to resolve their predicament, and on the other hand, may suggest that their emotional needs were met by the OHC in indirect ways. First, information seeking is frequently used as a coping response because it helps assess the degree of threat associated with a stressor, thereby reducing uncertainty in health care [69,70]. Second, although not directly soliciting emotional support, participants in the OHC voluntarily shared detailed accounts of their offline experiences while seeking information on self-managed withdrawal, pain management, and strategies for navigating health care environments. Emotional regulation in the form of venting feelings is within the underpinning of the transactional model of stress and coping [70,71]. Cathartic release and negation of offline frustration are also themes related to the negotiation of self-management support [35].

### Challenging the Scope of Self-Management Support

It is worth noting that self-management support interventions are typically developed by health care professionals to complement standard care. For example, self-management support for mental health typically focuses on patient education, medication adherence, relapse prevention, and coping strategies [28]. Among the 6 self-management support needs identified, the second need for patient education on how to scientifically reduce opioid dosage may challenge the realm of what is commonly accepted for self-management support in that self-managed withdrawal implies evading standard care, instead of complementing it. Self-managed withdrawal without professional supervision can be dangerous and should be discouraged. Here, we take the view that until there are enough specialized resources to treat pregnant women with opioid misuse and done so without legal penalization, there will always be women compelled to pursue self-managed withdrawal. Therefore, harm reduction via patient education as to how to safely taper is imperative.

### Policy Considerations

Although DATA was passed in 2000, women in the OHC still reported difficulty in obtaining MAT from their obstetricians’ office years later. To facilitate the initiation of recovery for pregnant women with opioid misuse, it is imperative to increase the number of obstetricians who are waivered buprenorphine prescribers and to increase the number of opioid treatment programs that cater to pregnant women. As of January 2020, approximately 10% of US physicians have received the DATA waiver [72]. Coverage in rural areas is particularly needed. For example, only 53% of outpatient buprenorphine prescribers accepted pregnant patients in Appalachian states as of 2017 [73]. Moreover, only 24% of opioid treatment facilities offer special programs for pregnant or postpartum women [1].

The popularity of self-management in the OHC highlights women’s needs for support in reducing their opioid dosage and their fear of seeking professional care. This indicates that legal penalization can be detrimental to the well-being of both the mother and child, as women avoid prenatal visits [4]. Many pregnant women presume that they will face negative consequences if they disclose drug use to their obstetricians, missing an important window in which to initiate recovery. Universal screening of substance use for pregnant women without legal implications may help dissolve the distrust between some patients and their providers.

### Technology Design for Harm Reduction

Although technology may not be able to directly change the medical and legal landscape, it can be used to tackle challenges faced by pregnant women with opioid misuse. Digital interventions have demonstrated small to modest effects in supporting people in recovery from SUDs [74,75]; however, only a minority of evidence-based self-help interventions have functional websites for general use [76]. A study on popular alcohol reduction apps found that common behavior change techniques employed facilitate self-recording, provide information on the consequences of excessive alcohol use, and provide feedback on performance [77]. As mentioned earlier, the social cognitive theory offers a framework to create positive behavior changes [68,69]. *Facilitation* and *self-regulation* are 2 concepts that are particularly applicable to better technological designs in the context of this study.

Facilitation refers to providing tools and resources to make new behaviors easier to establish. OHCs can facilitate (1) geo-specific information dissemination and (2) clinically validated tapering schedules for those who opt for self-management. Specifically, given the regional variations in law enforcement and resources of specialized care, organizing information specific to geographic areas may facilitate online discussions relevant to participants’ local environments. For those opting for self-managed withdrawals, we envision an online calculator that can account for women’s historical opioid dosage and gestational stage and generate a personalized tapering schedule based on current guidelines (albeit while expressing strong encouragement to bring one’s opioid use to the attention of their health care professionals).

Furthermore, self-regulation refers to controlling oneself through self-monitoring, goal setting, feedback, and the enlistment of social support. Although undergoing withdrawal, women in the OHC often lack structured social support that can hold them accountable and support them in their efforts to stay on track. Peer-led 12-step groups have been shown to improve accountability and recovery prospects for participants [78]. We envision OHC’s incorporating the structured aspect of effective peer support programs by creating an environment in which participants can perform daily check-ins, display badges of withdrawal progress, and easily reach out to peers for support and accountability.

### Limitations and Future Work

The findings of this study should be interpreted with limitations. First, the reported self-recovery trends are representative of those seeking help in the OHC. The percentage of self-managed withdrawal in the general population may be lower than that reported in this study, as those opting for self-management may have a greater propensity for participating in the OHC discussions to seek help. Our findings, however, are meaningful in better understanding the OHC population that appears to require relevant, clinically validated information. Second, only 200 posts for the OHC were analyzed. Thematic analyses are commonly applied to sociobehavioral studies using semistructured interviews with an average of 30 participants (SD 18.7) [79]. Qualitative studies of online content have varying sample sizes, usually ranging from 100 to 2000 [31,41,43,45,46,50,80]. We iteratively coded 200 posts and reached concept saturation within this number. In other words, had new concepts emerged during the annotation of the last 50 randomly sampled posts, we would continue sampling additional posts. Third, comments in response to the initiating posts by women with opioid misuse were not analyzed, as this study focused on the beliefs and actions of those attempting self-management at the moment of posting. Previous research shows that most comments in OHCs for chronic health conditions have an element of social support, primarily including validation and empathy [80]. In light of our observation on the imbalance of requests for informational and emotional support, future research should examine how the OHC audience responds to the posts. Fourth, qualitative analysis was performed on a cross-sectional rather than a longitudinal sample set. A future direction may be to follow OHC participants throughout the course of their pregnancy and postpartum to better understand the outcome of their proposed self-management work.

### Conclusions

OHCs provide vital self-management support for pregnant women with opioid use or misuse. Women pursuing self-managed dosage reduction are prone to misinformation and repeated relapses, which can result in extreme measures to avoid testing positive for drug use at labor. The study findings provide evidence for public policy considerations, including universal screening of substance use for pregnant women, emphasis on treatment rather than legal punishment, and further expansion of the DATA waiver training program. The improvement of online platforms that can organize geo-relevant information, dispense clinically validated withdrawal schedules, and offer structured peer support is envisioned for harm reduction among pregnant women who opt for self-management of opioid misuse.

## Appendix

Multimedia Appendix 1 Definitions related to opioid use and treatment.

Multimedia Appendix 2 List of 36 drug names related to opioid use.

## Abbreviations

CPS: Child Protective Service
DATA: Drug Addiction Treatment Act
MAT: medication-assisted treatment
NAS: neonatal abstinence syndrome
OHC: online health community
OUD: opioid use disorder
SUD: substance use disorder
