# Supplementary material for: Identifying Self-Management Support Needs for Pregnant Women With Opioid Misuse in Online Health Communities: Mixed Methods Analysis of Web Posts
Source: J Med Internet Res. 2021 Feb 4;23(2):e18296. doi: 10.2196/18296 (PMC7892281; doi:10.2196/18296)
Supplement: Multimedia Appendix 1 [file jmir_v23i2e18296_app1.docx]

## **Multimedia Appendix 1: Definitions related to opioid use and treatment**

**Opioid use disorder**

Opioid use disorder (OUD) is “a problematic pattern of opioid use leading to clinically significant impairment or distress” [1]. OUD is typically a chronic condition with high rates of relapse common during the treatment process [2]. The goal of OUD treatment is to achieve “recovery”, an overall improvement in quality of life as people in remission resume productive lives [3].

**Maternal use of opioids**

Maternal use of opioids can be divided into three types: prescription opioids, MAT opioids, and illicit opioids such as heroin [4]. It is still debated whether pregnant women with chronic pain can safely be prescribed an opioid medication, but the general consensus is that it can be considered - if appropriately indicated by symptoms and carefully evaluated against one’s history of substance use and the availability of alternative pain management therapies (e.g., nonopioid pharmacologic treatment, physical therapy, behavioral interventions) [5].

**Medication assisted treatment**

The American College of Obstetricians and Gynecologists recommends medication-assisted treatment (MAT) for prenatal opioid use disorder. MAT medications including methadone, buprenorphine and naltrexone are synthesized opioids that can bind with opioid receptors to curb cravings but have less addictive potential under medical supervision [6].

**Legal implication of opioid use in pregnancy**

The legal implications of drug use during pregnancy vary by regions in the U.S. Three states have explicit or interpretable statutes that treat exposing unborn children to drugs as a crime, and at least 45 states continue to attempt to prosecute women for using drugs during pregnancy [7]. Right to parental guardianship is judged on a case by case basis and varies by state procedures. Authorities rely on healthcare providers to inform maternal drug-use cases. The Keeping Children and Families Safe Act (2003) requires physicians to notify state Child Protection Services of any infant affected by illegal substances at birth or experiencing drug withdrawal. Although some states require healthcare providers to report suspected drug abuse during pregnancy [7], the ambiguity in law and its interpretation largely leaves healthcare providers on their own to decide whether to test, whom to test, and how to inform patients about their drug-testing policies.

### **References**

1. Module 5: Assessing and Addressing Opioid Use Disorder (OUD). Centers for Disease Control and Prevention. 2021.   URL: <https://www.cdc.gov/drugoverdose/training/oud/accessible/index.html> [accessed 2021-01-20]
2. Worley J. Recovery in substance use disorders: what to know to inform practice. Issues Ment Health Nurs 2017 Jan;38(1):80-91.
3. Commonly Abused Drugs Charts. National Institute on Drug Abuse. 2019.   URL: <https://www.drugabuse.gov/drugs-abuse/commonly-abused-drugs-charts> [accessed 2021-01-05]
4. Stover MW, Davis JM. Opioids in pregnancy and neonatal abstinence syndrome. Semin Perinatol 2015 Dec;39(7):561-565
5. Committee on Obstetric Practice. Committee Opinion No. 711: Opioid Use and Opioid Use Disorder in Pregnancy. Obstet Gynecol 2017 Aug;130(2):e81-e94.
6. Laslo J, Brunner J, Burns D, Butler E, Cunningham A, Killpack R, et al. An overview of available drugs for management of opioid abuse during pregnancy. Matern Health Neonatol Perinatol 2017 Feb 10;3(1):4
7. Propublica. How States Handle Drug Use During Pregnancy.   URL: <https://projects.propublica.org/graphics/maternity-drug-policies-by-state> [accessed 2021-01-20]
