# Supplementary material for: Identifying Self-Management Support Needs for Pregnant Women With Opioid Misuse in Online Health Communities: Mixed Methods Analysis of Web Posts
Source: J Med Internet Res. 2021 Feb 4;23(2):e18296. doi: 10.2196/18296 (PMC7892281; doi:10.2196/18296)
Supplement: Multimedia Appendix 2 [file jmir_v23i2e18296_app2.docx]

## **Multimedia Appendix 2: List of 36 drug names related to opioid use**

drugs = ['actiq', 'buprenorphine', 'codeine', 'demerol', 'dilaudid', 'dolophine', 'duragesic', 'duramorph', 'fentanyl', 'heroin', 'hydrocodone', 'hydromorphone', 'lofexidine', 'meperidine', 'methadone', 'methadose', 'morphine', 'ms+contin', 'naloxone', 'naltrexone', 'narcan', 'norco', 'opana', 'oxycodone', 'oxycontin', 'oxymorphone', 'percocet', 'percodan', 'propoxyphene', 'sublimaze', 'suboxone', 'subutex', 'tramadol', 'ultram', 'vicodin', 'zohydro']
